# Transcriptome Analysis and Resistance Identification of *bar* and *BPH9* Co-Transformation Rice

**DOI:** 10.3390/ijms26041762

**Published:** 2025-02-19

**Authors:** Sanhe Li, Changyan Li, Jianyu Wang, Lei Zhou, Bian Wu, Zaihui Zhou, Xiaolei Fan, Aiqing You, Kai Liu

**Affiliations:** 1 Hubei Key Laboratory of Food Crop Germplasm and Genetic Improvement, Laboratory of Crop Molecular Breeding, Food Crops Institute, Hubei Academy of Agricultural Sciences, Ministry of Agriculture and Rural Affairs, Wuhan 430064, China; lisanhe1@hbaas.com (S.L.); lichangyan@hbaas.com (C.L.); 202221107012051@stu.hubu.edu.cn (J.W.); zhoulei@hbaas.com (L.Z.); wubian@hbaas.com (B.W.); 2 College of Life Science and Technology, Huazhong Agricultural University, Wuhan 430070, China; zaihuizhou@mail.hzau.edu.cn; 3 Jiangsu Key Laboratory of Crop Genomics and Molecular Breeding, College of Agriculture, Yangzhou University, Yangzhou 225009, China; xlfan@yzu.edu.cn

**Keywords:** transgenic rice, *bar*, *BPH9*, resistance, transcriptome

## Abstract

Insect pests and weeds are the two major biotic factors affecting crop yield in the modern agricultural system. In this study, a brown planthopper (BPH) resistance gene (*BPH9*) and glufosinate tolerance gene (*bar*) were stacked into a single T-DNA cassette and transformed into an indica rice (*Oryza sativa* L.) line H23. The present study employed a gene stacking process that combines more than one gene/trait into an individual transgenic plant to meet the increasing cropping demands under complex conditions. The transgenic rice H23 (H23R) co-expressing *bar* and *BPH9* genes demonstrated both glufosinate tolerance and BPH resistance. We utilized transcriptome data to reveal the mechanism of *BPH9*-mediated brown planthopper resistance and to analyze the impact of exogenous transgenic fragments on upstream and downstream genes at insertion sites. The evaluation of insect resistance and glufosinate tolerance confirmed H23R as an excellent double-resistant transgenic rice. These findings indicate that H23R can satisfy insect management and weed control in the modern rice agricultural system. However, a deregulation study will help with prospective commercial planting.

## 1. Introduction

Rice is one of the most significant food crops for mankind, with the yield accounting for more than 40% of the total output of food crops. Two-thirds of the world’s population depend on rice as their staple food. The safe and stable production of rice is the key to guaranteeing world food security. Various adversities (such as pathogens, pests, drought, and salinification) affect the production process, causing great economic losses; meanwhile, population growth also poses an enormous challenge to food security [1,2,3]. The demand brought about by the continuous growth of the population can only be met by constantly updating varieties and assuring stability and increase in the grain yield [4,5].

Weeds in the field compete with crops for water, fertilizer, light energy, and growing space, directly influencing crop yield and quality. In addition, some weeds are intermediate hosts of crop pathogens and pests, and are one of the significant biological limiting factors for enhancing crop yields, so the effective control of weeds in the field is one of the important measures to increase grain yields. As the rural population migrates to cities at an accelerated rate, the mass production and mechanization of agricultural planting is a foreseeable trend, which makes traditional manual weeding impractical [6,7]. The popularization and use of herbicides can dramatically decrease the labor required for rice field management as well as labor intensity [8]. Since the first commercialization of transgenic crops in 1996, herbicide tolerance has always been the most important trait of transgenic crops. Currently, the trait of herbicide tolerance is widely used in soybeans, maize, oilseed rape, cotton, sugar beet, and alfalfa, with the area accounting for more than 60% of the transgenic crop area in the globe. The major functional genes are *PAT/bar* and *EPSPS* [9,10,11].

Rice planthoppers are the major pests that harm rice growth [12]. In recent years, the outbreak frequency of brown planthoppers has enhanced significantly, and the damage has become increasingly serious, severely threatening the superior quality and high yield of rice [13,14]. Now chemical control is still a crucial method to control brown planthoppers. However, many countries in Asia have reported that brown planthoppers are resistant to conventional organophosphorus and carbamate insecticides [15,16,17]. Utilizing the pest resistance of insect-resistant rice varieties is one of the most economical and valid ways to control brown planthopper populations. The breeding and application of insect-resistant varieties possess many advantages, such as less input, simple operation, no harm to natural enemies, no pollution to the environment, and coordinated use with other technologies [18]. Due to the development of molecular cloning technology, some major genes for resistance to brown planthoppers have been successfully cloned, which facilitates the study of the insecticidal mechanism of resistance genes and the application of resistance genes to breed insect-resistant varieties [19,20,21,22].

The breeding of resistant rice through direct transgenic methods has greater advantages than traditional breeding. Appropriate regulatory elements can be used to control the expression level of resistance genes, thereby achieving faster and better pest resistance effects [23,24,25,26]. At present, around 30 *BPH* genes have been identified, and multiple *BPH* genes have been cloned [27]. The *BPH9* gene is a special *NBS-LRR* gene located on the long arm of chromosome 12 of rice. The protein it encodes is located in the endomembrane system, including the NB (nucleotide-binding) region and LRR (Leucine-rich repeat) region [28]). The *BPH9* gene of Pokkali, an indica rice variety, was cloned. In some other insect-susceptible varieties, the *BPH9* gene region is divided by two transposable elements, leading to abnormal expression of the gene. *BPH9* may produce avoidance and antibiotic effects on brown planthoppers by activating the salicylic acid and jasmonic acid signaling pathways [29].

In this study, two constitutive expression promoters were used to drive *BPH9*, the insect-resistant gene, and *bar*, the glufosinate tolerance gene, to construct the expression vector BU9-3301 and transfer into the improved rice two-line hybrid parent H23 through Agrobacterium-mediated method. Finally, the insect-resistant and herbicide-resistant rice transformant H23R was obtained. The herbicide-resistant gene and the insect-resistant gene were constructed into the same expression vector. During the transformation stage, the herbicide-resistant gene can be used to screen transformants. The obtained positive transformants have the composite trait of insect resistance and herbicide resistance and can be directly used as breeding parents, which avoids the trait aggregation process via multi-generation backcrossing and shortens the breeding cycle. Meanwhile, the *bar* gene has a history of application for many years, receiving wide evaluation and recognition in terms of safety; the *BPH9* gene is derived from Pokkali, a cultivated indica rice variety, and the safety risk has been markedly decreased. Transcriptome data were used to further analyze the *BPH9*-mediated broad-spectrum and long-lasting resistance mechanism to brown planthopper in H23R. Thus, the commercial planting of the insect-resistant and herbicide-tolerant rice transformant H23R created in this study, which has the composite trait of insect resistance and herbicide tolerance, can not only decrease the use of pesticides, reduce production costs, and protect the environment, but also retrieve yield losses caused by pests and weeds, enhance rice production efficiency, and maintain food security, which has tremendous economic, environmental, and social benefits.

## 2. Results

### 2.1. Identification of Resistance to Herbicides and Brown Planthoppers in H23R

The *bar* gene transferred in this study makes the transformant H23R resistant to the herbicide glufosinate-ammonium, which is a phenotypic trait widely used in transgenic crops. The active ingredient of glufosinate-ammonium, L-PPT (L-phosphinothricin), is an inhibitor of glutamine synthetase. Because glutamine synthetase is involved in the synthesis system of enzymes required for the ammonia metabolism pathway, the inhibition of glutamate synthase will decline the efficiency of ammonia metabolism. The rapid accumulation of ammonia will lead to the disintegration of plant cell membranes and block photosynthesis, eventually causing plant wilt or even death.

**Figure 1:**
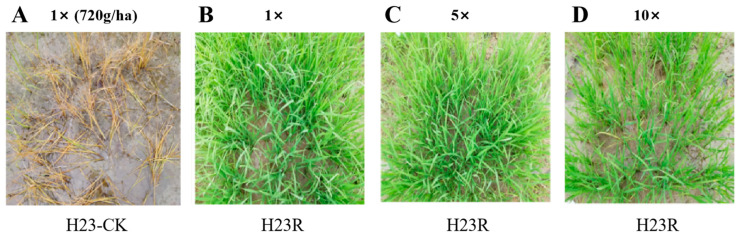
Identification of glufosinate-ammonium resistance of transgenic materials in the field. (**A**). H23-CK one week after 1× dose of the Basta treatment. (**B**). H23R one week after 1× dose of the Basta treatment. (**C**). H23R one week after 5× dose of the Basta treatment. (**D**). H23R one week after 10× dose of the Basta treatment.

This experiment evaluated the tolerance of transformant H23R to target herbicides by manually spraying 1, 5, and 10 times the recommended field dosages. The results showed that the transgenic plant H23R also had good tolerance to 10 times the target herbicide glufosinate-ammonium, with a tolerance grade of “excellent” (Figure 1). In future commercial production, the transgenic rice H23R and its derived lines with the trait of glufosinate-ammonium tolerance can be treated with the herbicide glufosinate-ammonium for weed control and killing, which provides an available measure for weed control in the field after planting transgenic rice and further guarantees the environmental safety of transgenic rice.

### 2.2. Resistance of H23R to Brown Planthopper During Seedling and Mature Stages

**Figure 2:**
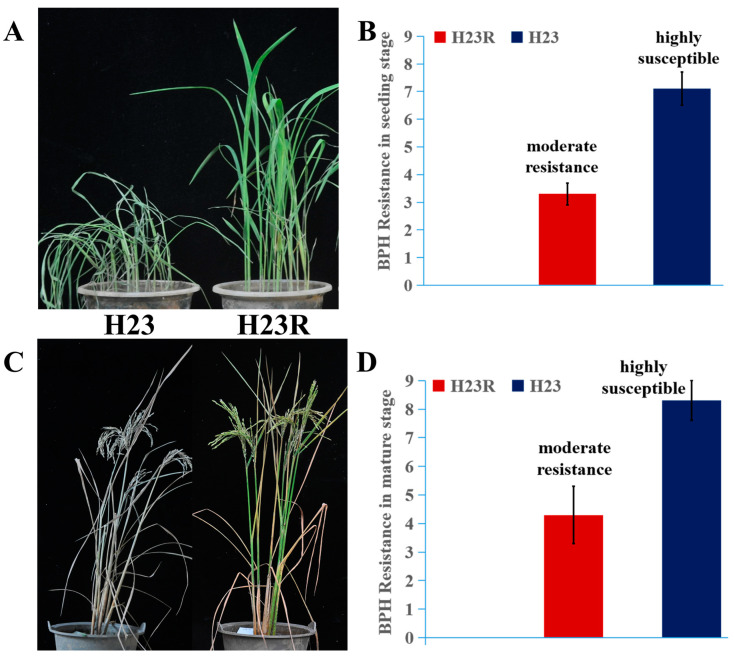
Resistance identification of brown planthopper. (**A**). Phenotypes of H23 and H23R resistant to brown planthopper at seeding stage. (**B**). The resistance levels of H23 and H23R to brown planthopper at the seedling stage. (**C**). Phenotypes of H23 and H23R resistant to brown planthopper at the mature stage. (**D**). The resistance levels of H23 and H23R to brown planthopper at mature stage. The methods and standards for identifying resistance of brown planthopper refer to Wang (2024) [25].

Brown planthoppers were inoculated at the seedling stage (2 weeks after sowing) and the mature stage (the peak tillering stage) for insect resistance identification. The results indicated that H23R showed excellent insect resistance compared to the control in both the seedling stage and the mature stage, demonstrating that H23R could be used in rice breeding for resistance to brown planthoppers (Figure 2).

Currently, the breeding of resistant rice varieties is one of the most economical and effective ways to control brown planthopper populations. The breeding and application of insect-resistant varieties have many advantages, such as less input, simple operation, no harm to natural enemies, no pollution to the environment, and coordinated use with other technologies. The use of transgenic methods to rapidly and efficiently breed new rice germplasm with composite traits of insect resistance and herbicide resistance will significantly enhance rice production efficiency.

### 2.3. Identification of PAT/bar and BPH9 Protein Content in H23R

**Figure 3:**
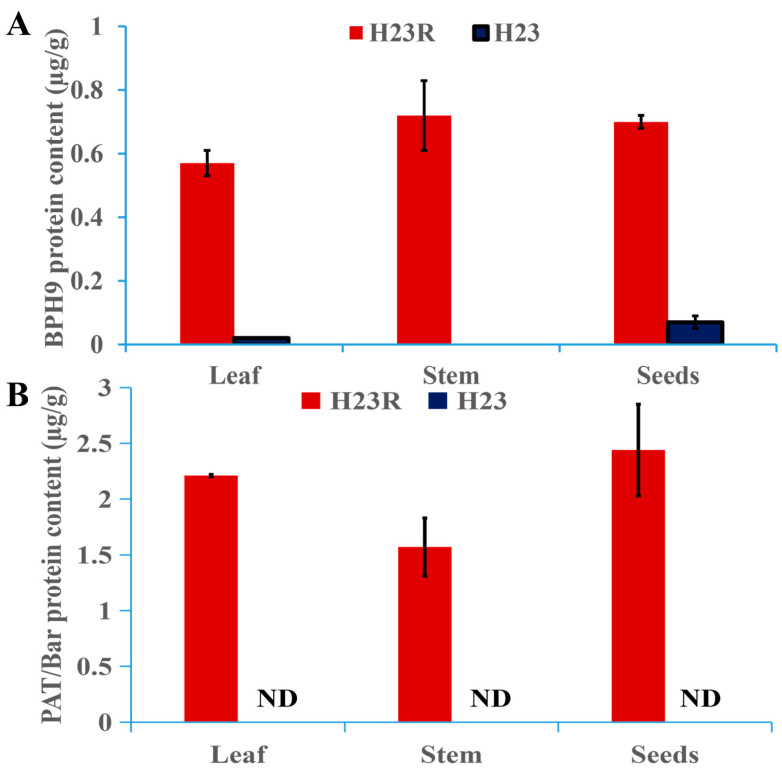
Target protein content in different rice tissues of T_2_ generation transformant H23R. (**A**). BPH9 protein content in different tissues. (**B**). PAT/bar protein content in different tissues.

Indirect ELISA was used to detect BPH9 protein. The results showed that there was essentially no BPH9 protein detected in the H23 wild-type control (the protein content of BPH9 in the leaves, stems, and seeds of H23 was 0.57 µg/g, ND, and 0.70 µg/g, the small amount detected in leaves and seeds may be the interference of endogenous homologous proteins). The protein content of BPH9 in the leaves, stems, and seeds of H23R was 0.57 µg/g, 0.72 µg/g, and 0.70 µg/g, respectively, as shown in Figure 3A.

The experiment adopted the PAT/bar Enzyme-linked immunoassay quantitative detection kit (Shanghai Youlong Biotech AA1041) to detect the PAT protein expressed by the bar gene. The results showed that no PAT/bar protein was detected in the H23 wild-type control. The protein content of PAT in leaves, stems, and seeds of H23R was 2.21 µg/g, 1.57 µg/g, and 2.44 µg/g, respectively, as shown in Figure 3B.

### 2.4. Transcriptome Differential Gene Analysis

**Figure 4:**
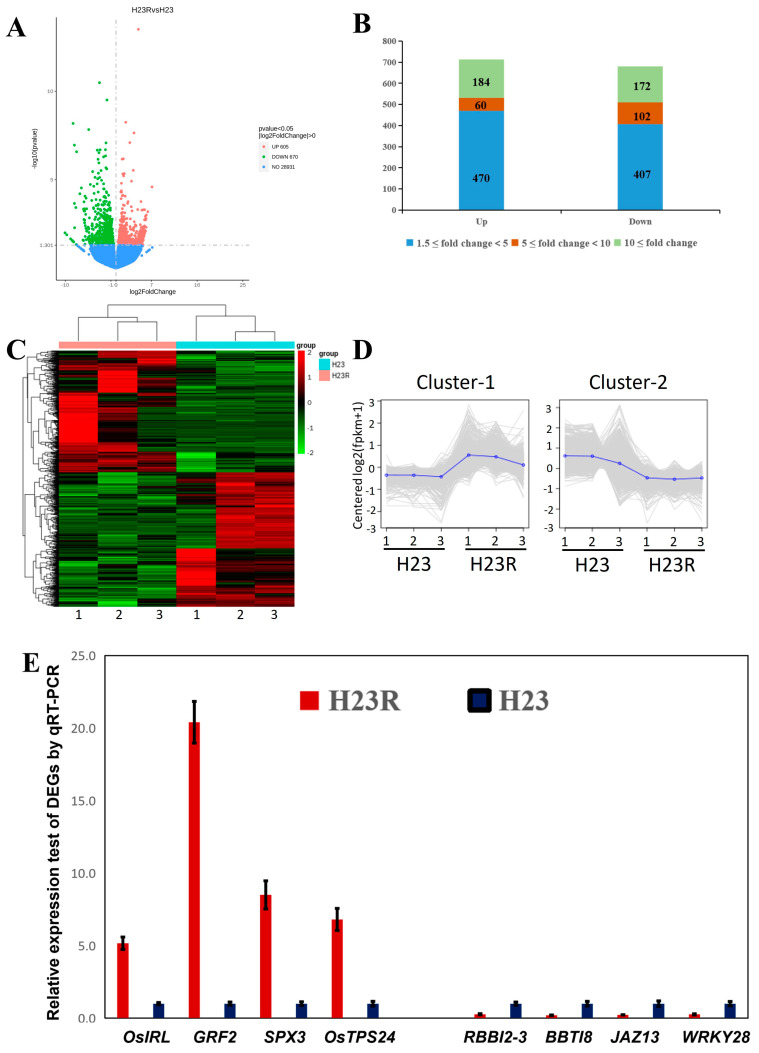
Differential expression gene analysis of H23R/H23 transcriptome. (**A**). Volcano plot of differentially expressed genes. (**B**). Differential fold distribution of differentially expressed genes. (**C**). Clustering of all the differentially expressed genes. (**D**). Expression pattern clustering of all the differentially expressed genes. (**E**). Q-RT PCR detection of partially differentially expressed genes.

By comparing the transcriptome data of the transgenic and control groups, significant differences in the expression of a total of 1275 genes were observed, of which 605 genes were up-regulated and 670 genes were down-regulated (Figure 4A). The fold of difference was divided into three intervals: 1.5- to 5-fold, 5- to 10-fold, and 10-fold and above. The data showed that the expression changes in most genes were within the range of 1.5- to 5-fold, indicating that the effect of *BPH9* transgene on rice gene expression was mainly moderate up-regulation or down-regulation. Nevertheless, many genes showed more than 10-fold significant differential expression, indicating that these genes played key roles in the *BPH9* transgenic rice (Figure 4B). These highly variable genes were possibly related to specific biological functions or stress response mechanisms of the *BPH9* transgenic rice.

In the three biological replicates of H23R and H23, the differential gene expression clustering was clear and obvious, indicating good biological repeatability and reliable data results (Figure 4C,D). Eight differentially expressed genes related to resistance were selected for qRT PCR expression level validation (Figure 4E). All differential expression gene information in the transcriptome can be found in Supplementary S3.

### 2.5. GO Analysis of H23R/H23 DEGs

**Figure 5:**
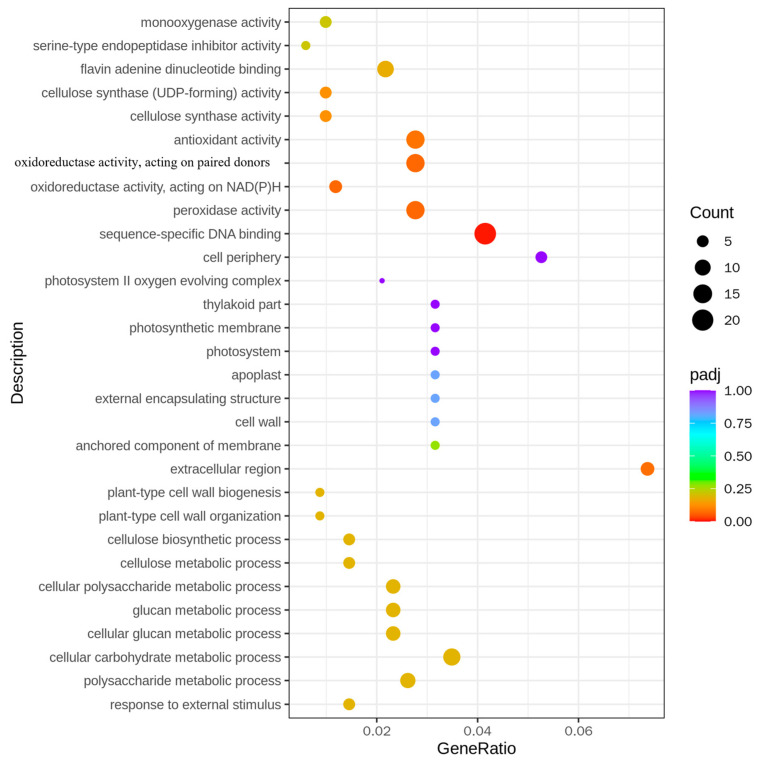
GO enrichment analysis of differentially expressed genes in H23R/H23.

In the analysis of all the differential genes, multiple significantly enriched biological processes and molecular functions were observed. For instance, genes relevant to cell wall synthesis such as “cellulose synthase activity” and “cellulose biosynthetic process” were significantly enriched, indicating that the transfer of the *BPH9* gene may affect the synthesis and organization of rice cell walls. Moreover, the enrichment of “antioxidant activity” and “peroxidase activity” showed that the expression of the *BPH9* gene possibly enhanced the antioxidant capacity of rice, which played an important role in resisting brown planthopper infestation. In terms of molecular function, the enrichment of “flavin adenine dinucleotide binding” and “oxidoreductase activity” indicated that the *BPH9* gene probably influenced the activity of enzymes related to energy metabolism and redox reactions. These changes might be connected with the resistance mechanism of rice to brown planthoppers (Figure 5).

In the analysis of up-regulated differential genes, the up-regulated expression of the genes related to energy metabolism and transport, such as “ATPase activity” and “proton-transporting ATP synthase complex”, was observed, indicating that the transfer of the *BPH9* gene strengthened the energy metabolism capacity of rice, which was necessary to support the physiological activities of resistance to brown planthoppers. The enrichment of “unfolded protein binding” and “dephosphorylation” showed that the transfer of *BPH9* gene possibly affected the folding and phosphorylation state of the protein, which might be related to the signal transduction pathway that regulated rice against the infestation of brown planthoppers (Supplementary Figure S1A).

In the analysis of down-regulated differential genes, the down-regulated expression of the genes related to photosynthesis, such as “photosystem II oxygen evolving complex” and “photosynthetic membrane”, was observed. This suggested that the transfer of the *BPH9* gene affected the photosynthetic efficiency of rice, which probably adjusted energy allocation to enhance resistance to brown planthoppers. In addition, the enrichment of “sequence-specific DNA binding” and “response to oxidative stress” showed that the transfer of the *BPH9* gene probably influenced the gene expression regulation and response to oxidative stress in rice. These changes might be connected with the adaptability of rice to the infestation of brown planthoppers (Supplementary Figure S1B).

### 2.6. KEGG Analysis of H23R/H23 DEGs

**Figure 6:**
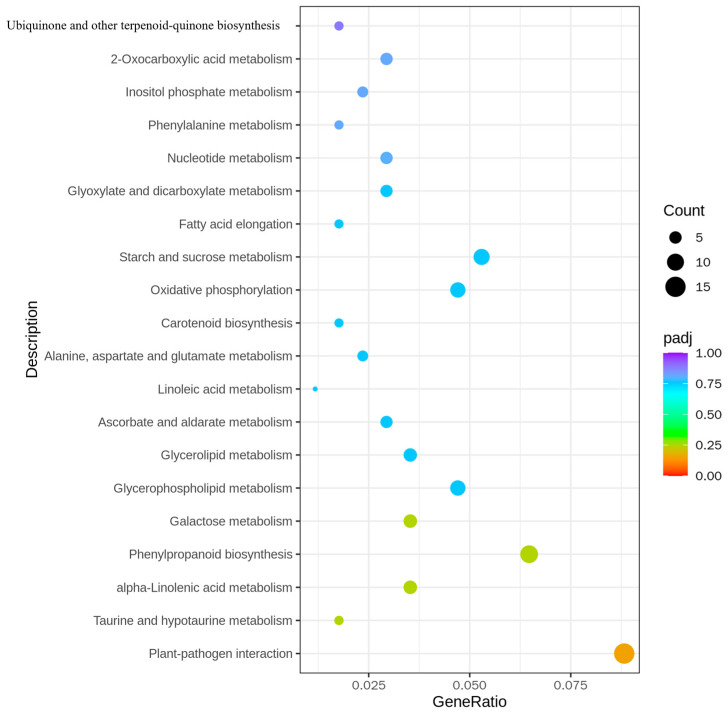
KEGG pathway enrichment analysis of differentially expressed genes in H23R/H23.

In the analysis of all the differential genes, significant enrichment of multiple metabolic pathways was observed. For instance, the enrichment of “Ubiquinone and other terpenoid-quinone biosynthesis” and “Oxidative phosphorylation” showed that the transfer of the *BPH9* gene may affect the energy metabolism and electron transport chain of rice. These changes may be connected with the enhancement of stress resistance and energy utilization efficiency of rice. The enrichment of “Carotenoid biosynthesis” and “Linoleic acid metabolism” may be related to the improvement of antioxidant capacity and membrane lipid fluidity of rice. The regulation of these metabolic pathways may help rice better adapt to environmental stress, including brown planthopper infestation. The enrichment of “Plant-pathogen interaction” showed that the transfer of *BPH9* gene may activate the defense mechanism of rice and strengthen the resistance to brown planthoppers, which may involve the activation of signal transduction pathways and the expression of defense-related genes (Figure 6 and Supplementary S4).

In the analysis of up-regulated differential genes, the enrichment of “Endocytosis” and “ABC transporters” may indicate that the transfer of the *BPH9* gene strengthens the membrane transport and material endocytosis capabilities of rice. This may help rice more availably deal with and eliminate harmful substances secreted by brown planthoppers. The enrichment of “Sesquiterpenoid and triterpenoid biosynthesis” may be related to the enhancement of secondary metabolism and synthesis of defense compounds in rice. These compounds possibly have anti-insect, anti-bacterial, or anti-fungal effects, thereby strengthening rice resistance. The enrichment of “Glycine, serine, and threonine metabolism” and “Glycerolipid metabolism” may be connected with the regulation of intracellular amino acid and lipid metabolism, which probably contribute to the maintenance of cellular function and structural integrity when rice is exposed to brown planthoppers infestation (Supplementary Figure S2A).

In the analysis of the down-regulated differential genes, the enrichment of “Photosynthesis-antenna proteins” and “Galactose metabolism” may indicate that the transfer of the *BPH9* gene affects photosynthesis and carbohydrate metabolism in rice. These changes may be connected with adjustments in energy allocation and metabolic demands to support resistance responses. The enrichment of “Sulfur relay system” and “Homologous recombination” may be connected with the regulation of the DNA repair and signal transduction pathways, which may help rice maintain genome stability and integrity in the face of brown planthopper infestation (Supplementary Figure S2B).

## 3. Discussion

Traditional weeding methods consume a mass of labor, while the cultivation of herbicide-resistant rice can sharply cut down the workload of manual weeding. This trait is particularly important today as labor costs are rising. By breeding herbicide-resistant rice varieties, herbicides can be used to control weeds during cultivation without damaging the growth of rice. The transgenic technology was used to make rice resistant to herbicides, enhance the competitiveness of crops with weeds in the field, and guarantee the growth stability and yield of crops. However, the long-term use of the same herbicide may cause weeds to develop resistance, so the scientific and rational use of herbicides is required. Breeding herbicide-resistant rice is of great significance to promoting the progress of agricultural science and technology, ensuring food security, and facilitating agricultural modernization. Simultaneously, it is necessary to comprehensively consider various factors, such as technology, environment, economy, and society, to guarantee healthy and orderly development. Glufosinate, a non-selective, broad-spectrum, highly efficient, and low-toxic organophosphorus herbicide, strongly inhibits the activity of glutamine synthetase (GS), an amino acid biosynthetic enzyme in bacteria and plants. GS plays a vital role in plant ammonia assimilation and metabolic regulation and is the only detoxification enzyme in plants, which can remove the toxicity of ammonia released by nitric acid reduction, amino acid degradation, and photorespiration. PAT protein is a phosphinothricin acetyltransferase (PAT, EC 2.3.1.183) which belongs to the acetyltransferase family. The substrate of PAT is phosphinothricin (PPT), which is the active ingredient of the herbicide glufosinate-ammonium. PAT can catalyze acetyl-CoA to acetylate the free amino group of PPT, the active ingredient of the herbicide glufosinate-ammonium, thereby detoxifying PPT, so that it cannot inhibit the activity of GS [11].

Since the Green Revolution, brown planthoppers have become the primary pest in global rice production. At present, most rice varieties are not insect-resistant, especially some major hybrid rice varieties even suitable for the growth and development of brown planthoppers, resulting in a widespread outbreak of brown planthoppers under the conditions of suitable climate. Brown planthoppers have developed resistance to dozens of the main insecticides (such as imidacloprid), which have special effects on pest control in the past decade, so the effectiveness of insecticide control has significantly declined. Despite the large-scale spraying of pesticides every year, serious yield losses of rice still occur in many places. Because the damage caused by brown planthoppers mostly occurs during the maturity and grain-filling stage of rice, the large-scale use of pesticides at this time also leads to severe pollution of the rice. Research has proven that using brown planthopper resistance genes to breed insect-resistant rice varieties is the most cost-effective method for comprehensive control of brown planthoppers. Combining transgenic technology with conventional breeding technology to breed brown planthoppers-resistant rice varieties and effectively control the occurrence and damage of brown planthoppers is an urgent task to guarantee green and efficient rice production and national food security.

*Bph9* encodes a CC-NB-NB-LRR protein containing two NB domains located in the intracellular membrane system of rice cells. *Bph9* is principally expressed in parenchyma cells around the xylem and sieve tubes, and is not induced by brown planthoppers feeding. After brown planthoppers feeding, the contents of SA, JA, and JA-Ile in *Bph9*-resistant materials are significantly enhanced. Gene chips, quantitative PCR, and exogenous hormone treatment have verified the role of SA and JA hormones in the insect resistance of *Bph9*. *Bph9* is located on the brown planthoppers-resistant gene cluster on chromosome 12, and there are seven other brown planthoppers-resistant genes in this interval. Sequence analysis shows that these eight genes are multiple allelic forms of the same gene and can be divided into four allelic types: *Bph1/9-1*, *Bph1/9-2*, *Bph1/9-7*, and *Bph1/9-9*. The research results on *Bph9* allelic variation and different resistance spectra will provide a reference for the mining and utilization of resistance allelic types [28,29].

The transcriptome data show that *BPH9* may be upstream of the defense response to brown planthoppers. The GO analysis shows that the transfer of the *BPH9* gene has an impact on cell wall synthesis, antioxidant capacity, photosynthesis, energy metabolism, and the signal transduction of rice. These changes may work together to strengthen rice resistance to brown planthoppers. The strengthening of the cell wall may directly impede the invasion of brown planthoppers, while the enhanced antioxidant capacity and energy metabolism may support the physiological defense response of rice in the face of invasion. In addition, the response to oxidative stress and the regulation of signal transduction may involve more intricate molecular mechanisms that jointly coordinate the resistance of rice to brown planthoppers; according to KEGG analysis, the transfer of the *BPH9* gene poses a wide impact on the metabolic pathways of rice, involving energy metabolism, antioxidation, activation of defense mechanisms, adjustment of photosynthesis and carbohydrate metabolism, regulation of signal transduction and DNA repair, etc. In particular, the enhancement of energy metabolism pathways may provide rice with more energy to support its defense responses. The improvement of antioxidant capacity is conducive to protecting rice from oxidative stress caused by brown planthoppers. Meanwhile, activated defense mechanisms may involve the recognition of pathogens, activation of defense gene expression, and production of defense compounds. Adjustments in photosynthesis and carbohydrate metabolism may be to optimize energy allocation in response to infestation. The regulation of signal transduction and DNA repair is of great importance to the maintenance of genome stability and the response to external stresses. Furthermore, enhanced membrane transport and material endocytosis capabilities probably contribute to the elimination or disposal of harmful substances secreted by brown planthoppers in rice. Enhanced synthesis of secondary metabolites may provide additional anti-insect, anti-bacterial, or anti-fungal effects. These analysis data also show the complicacy of the resistance mechanism mediated by *BPH9*. There are also many challenges for brown planthoppers to overcome rice resistance. *BPH9* is a resistance gene worthy of widespread promotion and use in production.

## 4. Materials and Methods

### 4.1. Generation of H23R Transgenic Rice Plants, Molecular Identification, and Screening of Single-Copy Homozygous Families

The plant expression vector used was BU9-3301, with a full length of 14,143 bp, and the T-DNA region including the left and right border sequences was 7760 bp. Its skeleton was pcambia3301, a vector commonly used in plant genetic engineering, with a kanamycin resistance gene expressed in bacteria. Agrobacterium-mediated genetic transformation was used to transfer it into H23. Single-copy homozygous positive family H23R was screened out in the T_2_ generation for further study via PCR positive detection and Southern hybridization copy number detection. The process of obtaining BPH and glufosinate-resistance rice is shown in Supplementary S1.

### 4.2. Identification of Resistance to Brown Planthoppers in H23R

1. Identification method of resistance to brown planthoppers at the seedling stage: The material to be identified was sown in the culture box. When the rice material grew to the two-leaf period, 7~8 brown planthopper nymphs with the age of 2~3 days old were added to each plant. When more than 90% of the control H23 died, each plant was scored and photographed according to the established grading criteria of 1 to 9. The final meaning value of each line was the resistance level of the material to be identified.
2. Identification method for brown planthoppers at the mature stage: The material to be identified was grown in the culture bucket until the peak tillering stage. Then, around 100 brown planthopper nymphs with an age of 2~3 days were added to each plant, and photos were taken two weeks later.

### 4.3. Identification of Herbicide Resistance

The non-transgenic control H23 and the transgenic family H23R were grown for about 3 weeks after sowing in the field. The H23R seedlings were sprayed with Basta herbicide at concentrations of 1× (720 g/ha glufosinate-ammonium), 5×, and 10×. The control H23 was sprayed with 1× glufosinate-ammonium. One week after applying glufosinate-ammonium, the growth status of the transgenic and control plants was evaluated and photographed.

### 4.4. Quantitative Protein Analysis of PAT and BPH9 in Different Parts of H23R and H23

The ELISA method was used to quantitatively measure the expression of different parts (seeds, stems, and leaves) of the transgenic rice.

The experiment used the PAT/bar enzyme-linked immunoassay quantitative detection kit (Shanghai Youlong Biotech AA1041, Shanghai, China) to detect the PAT protein expressed by the *bar* gene.

Indirect ELISA was adopted to detect BPH9 protein. The extracted sample solution and standards were coated on the microporous strip of the enzyme-labeled plate. When the BPH9 antibody was added, the samples/standards coated on the microporous strip captured it to form an antigen–antibody complex (Ag-Ab). The enzyme-labeled antibody (Ab-HRP) bound to the antigen–antibody complex to finally form an antigen–antibody-enzyme-labeled antibody complex (Ag-Ab-Ab-HRP), and then the color was developed by the enzyme-catalyzed TMB substrate reagent. After termination, the value was read by a microplate reader. The absorbance value of the sample is positively correlated with the BPH9 content, and the BPH9 content in the sample can be calculated by comparing it with the standard curve. The experimental method is described in Supplementary S2.

### 4.5. Transcriptome Analysis of H23R and Control H23

In this study, one leaf of H23R and H23 was taken as samples during the peak tillering stage (three biological replicates). Total RNA from each sample was extracted using TRIzolreagent (Thermo Fisher Scientific, Waltham, MA, USA) according to the manufacturer’s instructions and treated with RNase-free DNase I to remove genomic DNA contamination. After the determination of the quantity and quality using Agilent 2100 bioanalyzer (Santa Clara, CA, USA) and 1% denaturing gel electrophoresis, the RNA samples were sent to Smart Biotechnology Co., Ltd. (Tianjin, China) for RNA sequencing (RNA-seq).

High-quality reads were first aligned to the rice reference genome (https://rice.plantbiology.msu.edu/) using “HISAT2”. The results were subjected to “featureCounts v2.0.0” to obtain the read counts of all the samples. Differentially expressed genes (DEGs) were identified using “DESeq2 1.2.4R” (|log2(FC)| > 1 and Padj < 0.05). Gene Ontology (GO) (including biological process, cellular component, and molecular function) and KEGG pathway enrichment analyses of DEGs across the samples were performed through the clusterProfiler R package(4.14.4). Heatmap was illustrated using R/PHEATMAP and ComplexHeatmap, and the correlation coefficient matrices were generated and displayed using R/corrplot.

All differential expression gene information in the transcriptome can be found in Supplementary S3.

## 5. Conclusions

The insect-resistant and herbicide-tolerant rice H23R obtained in this study has the composite traits of insect resistance and herbicide tolerance. The planting of H23R can not only decrease the use of pesticides, reduce production costs, and protect the environment, but also reverse the yield loss caused by pests and weeds, improve rice production efficiency, and maintain food security, which has huge economic, environmental, and social benefits. *BPH9* is an allele of *BPH26*, while other BPH resistance genes in the cluster are alleles of *BPH9/26*. This gene locus exhibits extensive sequence diversity in rice germplasm resources. This discovery has significant implications for the co-evolution of host–pest interactions and the breeding of resistant varieties. Transcriptome analysis shows that corresponding to the phenotype of the high resistance to brown planthoppers of *BPH9*, its resistance mechanism involves multiple different signaling pathways and biological processes, which is beneficial to delaying the overcoming of brown planthoppers in production. *BPH9* is also a significant supplement to similar resistance gene resources in the existing production.

## Data Availability

The original contributions presented in the study are included in the article and Supplementary Materials, further inquiries can be directed to the corresponding authors.

## Supplementary Materials

The following supporting information can be downloaded at https://www.mdpi.com/article/10.3390/ijms26041762/s1.
