# Supplementary material for: Transcriptome Analysis and Resistance Identification of bar and BPH9 Co-Transformation Rice
Source: Int J Mol Sci. 2025 Feb 19;26(4):1762. doi: 10.3390/ijms26041762 (PMC11855366; doi:10.3390/ijms26041762)
Supplement: Supplementary file 1 [file ijms-26-01762-s001.zip › Supplementary Figures S1 and S2.pdf]

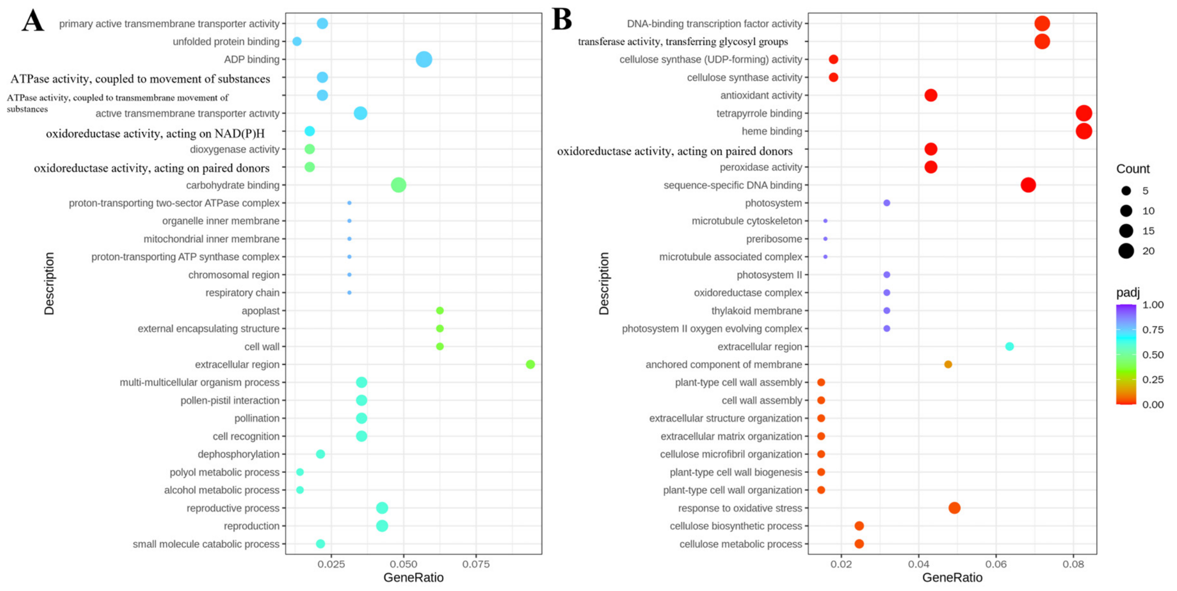

Supplementary Figure S1. GO enrichment analysis of up-regulated and down-regulated differential genes in H23R/H23.

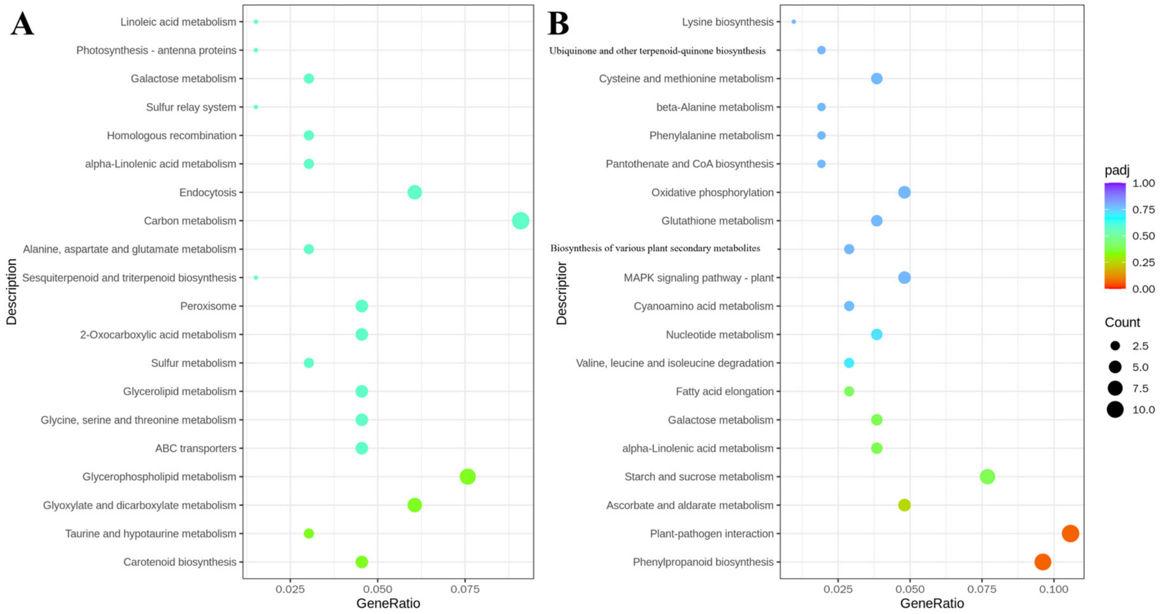

Supplementary Figure S2. KEGG pathway enrichment analysis of up-regulated and down-regulated differential genes in H23R/H23.
