# Supplementary material for: Transcriptome Analysis and Resistance Identification of bar and BPH9 Co-Transformation Rice
Source: Int J Mol Sci. 2025 Feb 19;26(4):1762. doi: 10.3390/ijms26041762 (PMC11855366; doi:10.3390/ijms26041762)
Supplement: Supplementary file 1 [file ijms-26-01762-s001.zip › Supplementary S1 The process of obtaining BPH and glufosinate-resistance Rice.pdf]

The process of obtaining BPH and glufosinate-resistance Rice

1) Construction of BPH9 and Bar co-expression vector and transformation

In this study, to generate transgenic rice, the plasmid BU9-3301 (**Figure 1**) was transferred to the indica rice sterile line H23 via modified *Agrobacterium*-mediated transformation to generate transgenic rice. The BU9-3301 vector was created utilizing pCAMBIA3301 as the backbone and homologous recombination of the *BPH9* gene obtained from BPH-resistance variety Pokkali.

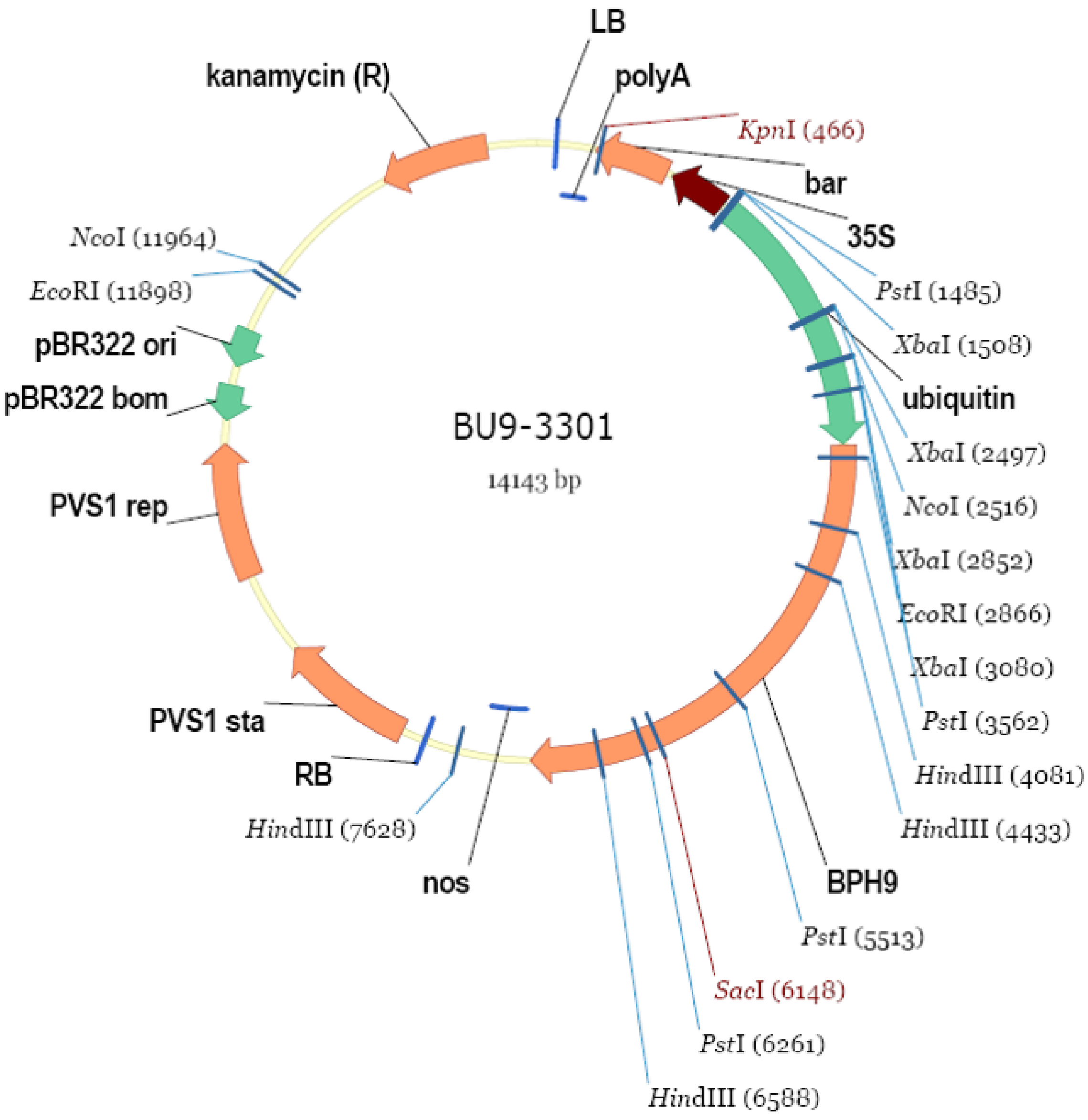

Figure 1 Structure of plasmid BU9-3301

2) Genotype identification of 20 T_0 generation transgenic lines

The T_0 progeny plants were grown in a greenhouse with a photoperiod of 12:12 h light:dark at 28/24 ℃ and 70% relative humidity. The putative transgenic lines were confirmed via PCR using the primers specific for *bar* and *BPH9* genes, and 20 positive rice transgenic lines were obtained (**Figure 2**).

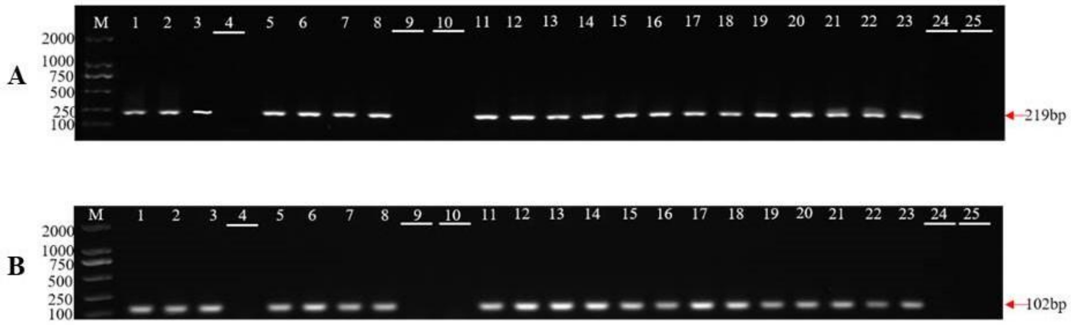

Figure 2 Identification of positive transgenic line

PCR analysis was performed to detect 20 positive transgenic lines with *BPH9* (A) and *bar* (B) gene specific primers in T0 progeny. M represents DNA ladder.

3) Search for single copy transgenic line H23R and tail-PCR analysis for T-DNA integration site

Southern blot analysis was performed to determine the copy number and stability of the exogenous genes in H23R (a quasi-commercial transformation event has passed the production trial of China's genetically modified safety evaluation and is currently applying for the final safety certificate), using H23 as the control. EcoRI and XbaI were selected for the bar gene to digest the genomic DNA. EcoRI has two restriction sites in the BU9-3301 vector, one in the T-DNA insert and the other on the backbone (Figure 3A).

Furthermore, SacI and XbaI were chosen to detect the BPH9 gene in H23. SacI and XbaI have one and four restrictions sites, respectively, in the T-DNA insert. Bands of correct size appeared after SacI or XbaI digestion in the positive control BU9-3301 vector (Figures 3B).

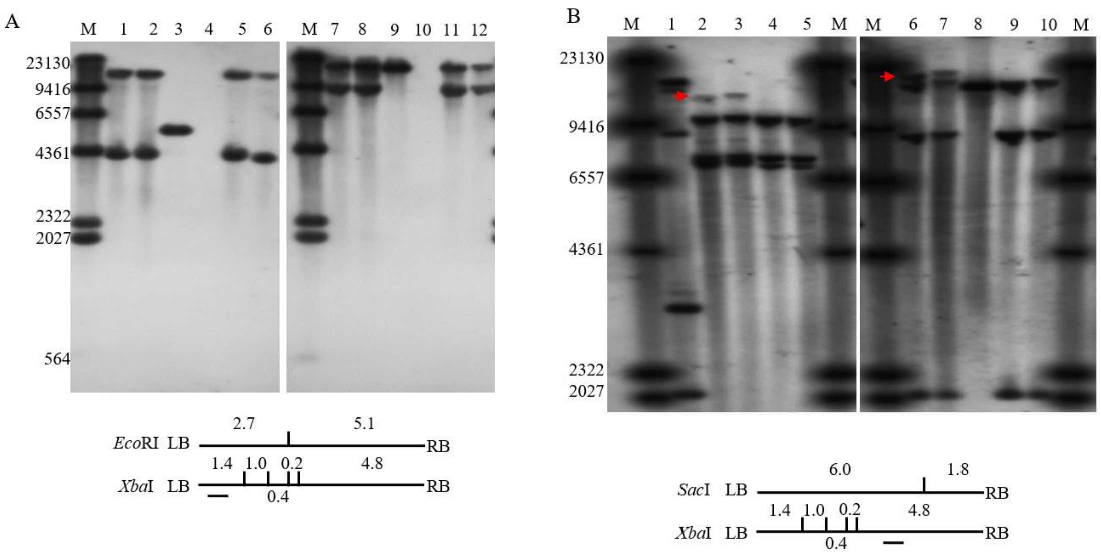

Figure 3. Southern blot analysis of bar gene and BPH9 gene in H23R.

A: Southern blot result with EcoRI and XbaI digestion for bar gene; **B:** Southern blot with SacI and XbaI digestion for BPH9.

The T-DNA region inserted into the exon of gene *LOC_Os03g47140* in transgenic plant. Red arrow indicates the insert location (Figures 4).

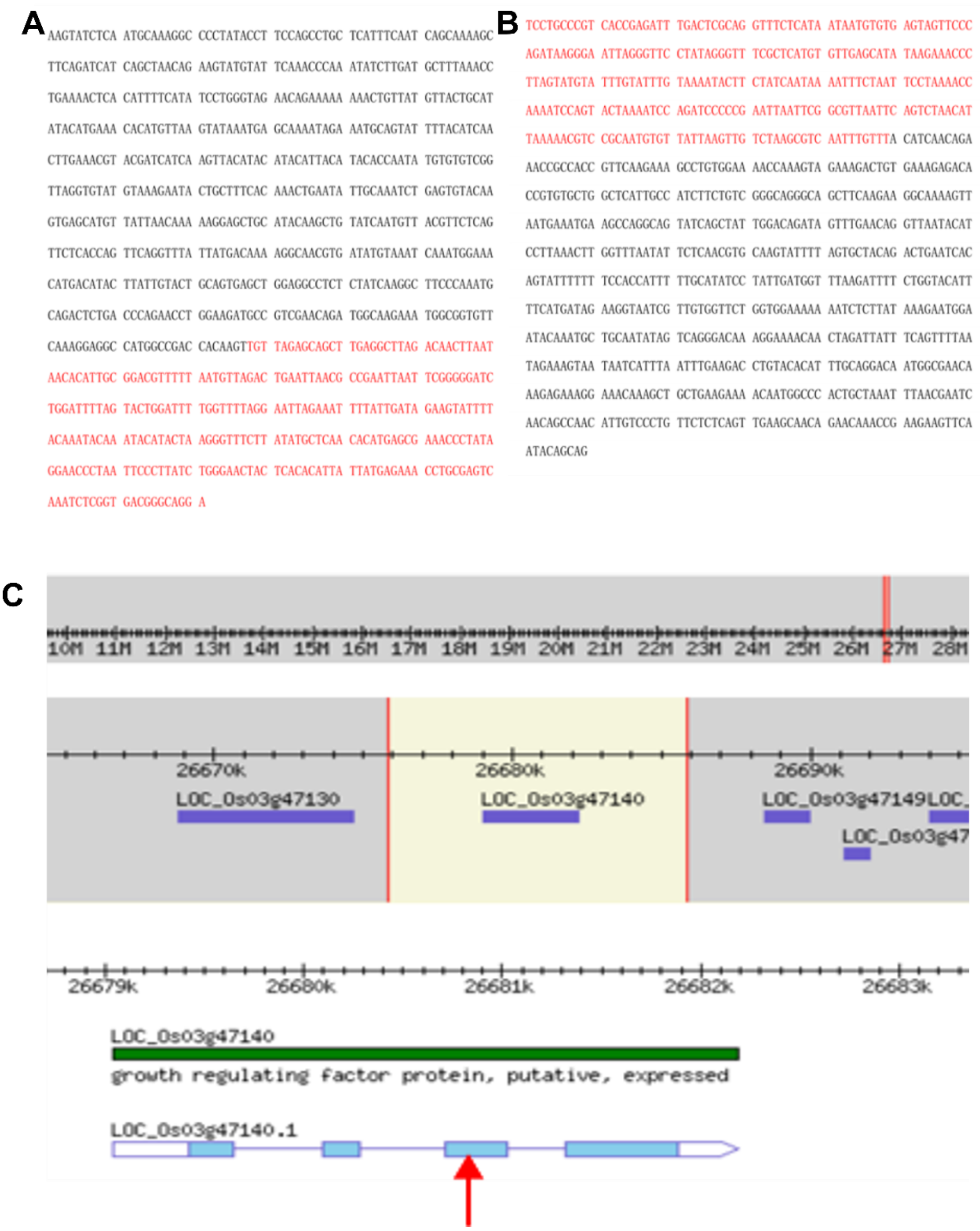

Figure 4 Isolated flanking sequence and schematic of the T-DNA insertion site in H23R.

A: left flanking sequence; **B:** right flanking sequence; **C:** the T-DNA insertion site in H23R.

4) Agronomic trait analysis of H23R

To evaluate the agronomic trait, the transgenic line H23R and the nontransgenic line H23 were grown in a greenhouse. There was no significant difference between H23R and H23 in plant height, effective spike number, spike length, total number of grains

per spike, seed setting rate, thousand grain weight, or yield (Table 1). The results showed that the insertion of T-DNA had no effect on the agronomic characteristic in the H23R.

Table 1 Agronomic trait analysis of H23R

Material	Plant height (cm)	Effective spike number	Spike length (cm)	Total number of grains per spike	Seed setting rate (%)	Thousand grain weight (g)	Yield (kg/acre)
H23R	117.9±1.1 a	14.9±0.8 a	20.4±2.3 a	236.7±17.9 a	69.1±2.9 a	22.9±1.2 a	572.3±37.2 a
H23	119.3±1.3 a	13.3±2.3 a	19.6±1.2 a	251.3±11.5 a	63.8±3.0 a	23.1±1.8 a	566.0±23.0 a

Statistical significance was analyzed using Duncan's multiple range test with criterion $\alpha = 0.05$
