# Supplementary material for: Transcriptome Analysis and Resistance Identification of bar and BPH9 Co-Transformation Rice
Source: Int J Mol Sci. 2025 Feb 19;26(4):1762. doi: 10.3390/ijms26041762 (PMC11855366; doi:10.3390/ijms26041762)
Supplement: Supplementary file 1 [file ijms-26-01762-s001.zip › Supplementary S2 Enzyme-linked immunosorbent acid (ELISA) was used to detect the expression of exogenous genes.pdf]

Enzyme-linked immunosorbent acid (ELISA) was used to detect the expression of exogenous genes

PAT/bar protein ELISA detection method

Technical principle

This kit uses a dual-antibody sandwich ELISA to detect the PAT/bar transgenic components in the samples. Monoclonal antibodies specific to PAT/bar are pre-coated on microstrips of the microplate plates. When the PAT/bar-containing sample is added, the pre-coated PAT/bar antibody on the microstrip captures it to form an antibody-antigen complex (Ag-Ab), and then an enzyme-labeled antibody (Ab-HRP) is added to bind to the antigen-antibody complex to form an antibody-antigen-enzyme-labeled antibody complex (Ab-Ag-Ab-HRP), and then the enzyme-catalyzed TMB substrate reagent is developed, and the microplate reader is read after termination. The absorbance value of the sample is positively correlated with the amount of PAT/bar it contains, and the amount of PAT/bar in the sample can be calculated by comparing it to the standard curve.

Materials and reagents provided

Enzyme label 96-well foldable plate, PAT/bar calibrator (8ng), 11× enzyme standard concentrate, enzyme diluent, chromogen, stop solution.

Equipment and instruments

Microplate reader (450 nm), incubator, shaker, vortex instrument, centrifuge, polystyrene centrifuge tube: 1.5 mL, micropipette: single channel 20 μL~200 μL, 100 μL~1000 μL, multichannel 250 μL, fresh ultrapure or deionized water.

Preparation of solutions

Preparation 1: Washing working solution

20 × of the concentrated washing solution was diluted with deionized water at a volume ratio of 1:19 (1 part of 20× concentrated washing solution + 19 parts of deionized water) for the washing of the microplate plates, and the washing solution could be stored at 4°C for one month.

Preparation 2: Calibrator preparation

Dissolve the PAT/bar calibrator with 1 mL of sample extraction and prepare the calibrator buffer at 0.5 ppb, 1 ppb, 2 ppb, and 4 ppb, respectively.

After diluting the standard solution, please protect it from light and store it at -20°C for 14 days.

Preparation 3: Enzyme label working solution

Dilute 11 × Enzyme Standard Concentrate in a 1:10 volume ratio (1 part of 11× Enzyme Standard Concentrate + 10 parts of enzyme dilution) with an enzyme dilution, e.g., 180 μL of 11× Enzyme Standard Concentrate + 1.8 mL of enzyme dilution, which can be used for 16-well assays.

Sample preparation method

(1) Take 0.1 g of crushed plant sample;

(2) Put it into a 1.5 mL centrifuge tube, add 1 mL of sample extraction solution, shake and mix for 5 minutes;

(3) Centrifuge at 4000 rpm for 3 min;

(4) Take 100 μL of supernatant and dilute 100 or 1000 times for analysis.

Detection steps

(1) Add 100 μL standard/sample extract) to each well, and react at 25°C for 45 min;

(2) Wash the plate, add 100 μL antibody working solution, and react at 25°C in the dark;

(3) Wash the plate, add 100 μL of enzyme label working solution, and react at 25°C in the dark for 30 min;

(4) Wash the plate, add 100 μL chromogenic agent, and react at 25°C in the dark;

(5) Add 100 μL stop solution, microplate reader 450 readings.

Judgment of the result

Calculation of the absorbance value of the standard, the average of the absorbance values of the standard or sample (double wells) minus the absorbance value of the sample dilution (background).

Drawing and calculation of standard curves

The absorbance value of the standard was used as the ordinate, and the concentration of the PAT/bar standard (ppb) was used as the abscissa, and the standard curve was plotted. The absorbance value of the sample is substituted into the standard curve, and the concentration corresponding to the sample is read out from the standard curve.

BPH9 protein ELISA detection method

Technical principle

Indirect ELISA was used to detect BPH9 protein components in samples. When BPH9

antibody is added, the sample/standard coated on the microstrip is captured to form an antigen-antibody complex (Ag-Ab), and the enzyme-labeled antibody (Ab-HRP) binds to the antigen-antibody complex to form an antigen-antibody complex (Ag-Ab-HRP), and finally forms an antigen-antibody-enzyme-labeled antibody complex (Ag-Ab-HRP), and then the color is developed by the enzyme-catalyzed TMB substrate reagent, and the microplate reader is read after termination. The absorbance value of the sample is positively correlated with the amount of BPH9 protein it contains, and the amount of BPH9 protein in the sample can be calculated by comparing it with the standard curve.

Materials and reagents provided

Enzyme label 96-well foldable plate, BPH9 calibrator, 11× concentrated enzyme label, enzyme diluent, chromogen, stop solution.

Equipment and instruments

Microplate reader (450 nm), incubator, shaker, vortex instrument, centrifuge, polystyrene centrifuge tube: 1.5 mL, micropipette: single channel 20 μL~200 μL, 100 μL~1000 μL, multichannel 250 μL, fresh ultrapure or deionized water.

Preparation of solutions

Preparation 1: Washing working solution

20 × of the concentrated washing solution was diluted with deionized water at a volume ratio of 1:19 (1 part of 20× concentrated washing solution + 19 parts of deionized water) for the washing of the microplate plates, and the washing solution could be stored at 4°C for one month.

Preparation 2: Calibrator preparation

Dissolve the BPH9 calibrator with 1 mL of sample extract and make standard dilutions at 0.0625, 0.125, 0.25, 0.5, and 1 ppm, respectively.

After diluting the standard solution, please protect it from light and store it at -20°C for 14 days.

Preparation 3: Enzyme label working solution

Dilute 11 × Enzyme Standard Concentrate in a 1:10 volume ratio (1 part of 11× Enzyme Standard Concentrate + 10 parts of Enzyme Dilution), e.g., 180 μL of 11× Enzyme Standard Concentrate + 1.8 mL of Enzyme Dilution, which can be used for 16-well assays.

Sample preparation method

(1) Take 0.1 g of crushed plant sample;

(2) Put it into a 1.5 mL centrifuge tube, add 1 mL of sample extraction solution, shake and mix for 5 minutes;

(3) Centrifuge at 4000 rpm for 3 min;

(4) Take 100 μL) and dilute 10 times after supernatant for analysis.

Detection steps

(1) Protein/sample 100 μL, 37°C reaction for 3 h;

(2) Wash the plate 3-5 times, pat dry, add BPH9 protein rabbit polyantibody, and react at 25°C in the dark for 45 min;

(3) Wash the plate 3-5 times, pat dry, add rabbit secondary antibody, and react at 25°C in the dark for 30 min;

(4) Wash the plate 3-5 times, pat it dry, add 100 (L chromogenic agent, 25 °C and avoid light for 15 min;

(5) Add 100 μL stop solution, and read the OD value with a microplate reader 450.

Result calculation

The OD value of the standard protein was used as the abscissa, and the concentration of the standard was used as the ordinate, and the standard curve was plotted (the average value of the replicated samples was taken). The OD value of the sample to be measured is brought into the standard curve to calculate its concentration.

If the OD value of the sample to be tested is higher than the upper limit of the standard curve, it should be diluted appropriately and retested, and the calculated concentration should be multiplied by the dilution factor.
